# Editorial: Acetogens - from the origin of life to biotechnological applications

**DOI:** 10.3389/fmicb.2023.1186930

**Published:** 2023-04-20

**Authors:** Mirko Basen, Volker Müller

**Affiliations:** ^1^Microbiology, Institute of Biological Sciences, University of Rostock, Rostock, Germany; ^2^Department of Molecular Microbiology and Bioenergetics, Institute of Molecular Biosciences, Johann Wolfgang Goethe University, Frankfurt, Germany

**Keywords:** acetogens, origin of life, synthesis gas fermentation, Wood-Ljungdahl pathway, CODH/ACS, acetyl-CoA pathway, CO_2_ fixation, hydrogenase

Acetogenic bacteria are a fascinating group of strict anaerobic bacteria characterized by a pathway, the Wood-Ljungdahl pathway (WLP), in which two molecules of carbon dioxide (CO_2_) are reduced and condensed to one molecule of acetyl-CoA. This product of CO_2_ fixation is the key intermediate in anabolism since it is the precursor of every cellular component in acetogens. Acetyl-CoA also is the key intermediate in catabolism, where it is further converted to acetate via acetyl-phosphate, which is the only ATP-generating reaction of CO_2_ reduction. Since one ATP is consumed in the activation of the intermediate formate in the WLP, the overall ATP gain is zero. Therefore, acetogens need an additional way to conserve energy. Hydrogen is used as reductant in the WLP. It is activated by hydrogenases, and subsequently, the electrons are transferred to electron carriers (e.g., NAD^+^, ferredoxin, NADP^+^) that differ from species to species by an array of soluble and membrane-bound transhydrogenases. The reduced electron carriers in turn provide the electrons to the WLP. The secret of energy conservation in acetogens growing on H_2_+CO_2_ is the excess of reduced ferredoxin (Fd_red_) from H_2_ oxidation, that is in turn re-oxidized by membrane-bound enzymes complexes, the Rnf complex or Ech hydrogenases. These enzymes at the same time provide NADH, NADPH or H_2_ to the WLP, and conserve energy by sodium ion or proton translocation. Of the known seven pathways of CO_2_ fixation, the WLP has the most favorable ATP balance (one ATP needed for the synthesis of acetyl-CoA) and therefore, is considered the oldest biochemical pathway on Earth, and the starting point for the synthesis of living matter from CO_2_ and H_2_ or carbon monoxide (CO), gaseous compounds present on Early Earth. Indeed, acetogens grow by acetogenesis from H_2_ + CO_2_ and they have additional, chemiosmotic mechanisms of energy conservation that ensure a net synthesis of ATP, as mentioned above. CO_2_ reduction to acetyl-CoA with H_2_ as reductant is also performed by methanogenic archaea in their anabolism, but in catabolism, the methyl group is released as methane. William Martin discusses the possible evolution of methanogenesis and acetogenesis, highlights similarities and differences and critically elaborates on the question whether the WLP indeed was the first CO_2_ fixation pathway. Along the same lines, Lemaire et al. discuss on a more biochemical level the different strategies involved in CO_2_ fixation in acetogens and methanogens, with a particular focus on energy conservation mechanisms. They use the editors' favorite terms of “energy extremophiles” for organisms that only conserve a fraction of an ATP per substrate turnover. Indeed, acetogens grow close to the thermodynamic limit of life (ΔG_0_' < −30 kJ mol^−1^). The authors highlight the importance and mechanistics of electron bifurcation in the energy metabolism of both groups. This mechanism links redox homeostasis e.g., the concomitant reduction of NAD^+^and Fd by electron bifurcating hydrogenase, to energy conservation, since Fd_red_ can be used for chemiosmotic energy conservation, or to drive reactions at low potential such as CO_2_ reduction, “saving ATP”. Methanogenesis is thermodynamically preferred over acetogenesis and thus methanogens outcompete acetogens during growth on H_2_ + CO_2_. Fu et al. demonstrate a preference of chemolithotrophic acetogenesis at 15°C and 50°C while hydrogenotrophic methanogenesis dominated at 30°C. Under certain conditions, however, acetogens prevail. Fazi et al. report, that in a natural environment with a high CO_2_ concentration acetogens are enriched and outcompete methanogens. Methanogens are archaea and acetogens are bacteria, but use the same pathway for CO_2_ fixation to acetyl-CoA, the WLP. The long-standing assumption that acetogenesis should also be present in archaea was found to be true in the last decade. Here, Loh et al. discuss the WLP in a bathyarchaeon isolated from the termite hind gut that is different from the bacterial one. Different gas availabilities influence the prevalence of certain acetogens as well. In her review, Philips discusses the different mechanisms of cathodic electron uptake by acetogens in the light of thermodynamics and kinetics of H_2_ uptake. She suggests that the ability of acetogens to thrive on cathodes correlates with the ability to maintain low H_2_ partial pressures.

The WLP is not only used by acetogens for CO_2_ reduction but also for oxidation of more reduced carbon compounds such as formate or methyl groups (derived, for example, from methanol). And some acetogens can even oxidize acetate to CO_2_, and produce H_2_. One such organisms is *Thermoacetogenium phaeum*. Keller et al. have addressed the enzymes involved in the reverse WLP and the energetics of acetate oxidation; they present interesting data on reverse electron transport in energy coupling, and metabolic schemes for the bioenergetics of acetate oxidation.

The WLP enabled growth of first life forms on Earth and is used by many bacteria for chemolithoautotrophic growth on H_2_ + CO_2_ and by some for chemoorganoheterotrophic growth on acetate. However, the metabolism of acetogens is much more interesting and diverse. They can grow on sugars, carboxylic acids, aldehydes as well as on primary, and secondary alcohols. Many of these substrates can only be oxidized by acetogens because electrons derived from the oxidation are transferred to CO_2_ that is used as electron acceptor; under these conditions the WLP acts as an electron sink. This was experimentally demonstrated by Jain et al. They used a novel genetic system to knock out the genes encoding a key enzyme in the WLP in the thermophilic acetogenic bacterium *Thermoanaerobacter kivui* and found that cells are no longer able to grow on organic substrates. Addition of formate restored growth, reinforcing the importance of the WLP for redox balancing. Similarly, Moon et al. show that oxidation of the reduced sugar alcohol mannitol is dependent on external CO_2_ as electron acceptor.

The diverse metabolism of acetogens is a consequence of their phylogenetic diversity. Acetogenesis is not a phylogenetic trait but found in many different phylogenetic lineages. Valk et al. found acetate formation form galacturonate by a new species of the family *Lachnospiraceae* using the WLP; however, the metagenome sequences lack a canonical acetyl-CoA synthase/CO dehydrogenase (ACS/CODH) gene cluster and the authors suggest a novel ACS/CODH in this species. Merino et al. isolated the microbiota of a hot spring in Japan and found novel actinobacteria. Based on metabolic pathway predictions, these actinobacteria are anaerobes, capable of glycolysis, dissimilatory nitrate reduction and CO_2_ fixation via the WLP. Even within the genus *Moorella*, there are surprises. Redl et al. provide a comprehensive genome analysis of all *Moorella* strains and question the difference of the previously acknowledged strains *Moorella thermoacetica* and *Moorella thermoautotrophica*.

Acetogens are prime candidates as production platforms in a CO_2_-based economy, but the products that can be formed from CO_2_ may be limited due to energetic constraints. Some acetogens can use electron acceptors other than CO_2_ such as nitrate or dimethylsulfoxide, and the simultaneous use of two different electron acceptors may chance carbon flow to more reduced end products. Klask et al. report that addition of nitrate not only enhances growth of *Clostridium ljungdahlii* but also shifts the product spectrum to ethanol. Along those lines, Zhu et al. report that addition of carbon monoxide increases the cellular ATP level and thus enables ethanol formation in *Clostridium ljungdahlii*. The redox potential of CO allows for more ferredoxin reduction, the fuel of chemiosmotic energy conservation, leading to more ATP. CO, however, is highly toxic, and toxicity of CO on a whole cell level is poorly understood. Kang et al. report on the *Adaptive Laboratory Evolution of Eubacterium limosum ATCC 8486 on Carbon Monoxide*. Genome analyses of the evolved strain revealed mutations in the ACS/CODH and when these mutations were generated in the wild type, the same phenotype was observed, highlighting the role of ACS/CODH in CO toxicity. Arantes et al. isolated a novel strain of *Acetobacterium wieringae* able to grow on CO, a trait not common for *Acetobacterium* species. Genome analyses suggest the formate dehydrogenase as reason for the apparent CO insensitivity.

In summary, the 16 publications in this Research Topic are as diverse as the physiological group itself, reflecting the importance of acetogens for understanding fundamental and ancient principles of Life that are as well widespread in nature and of biotechnological interest.

## Author contributions

MB and VM wrote and edited the editorial. All authors contributed to the article and approved it for publication.

